# A Comparison of Different Operating Systems for Femtosecond Lasers in Cataract Surgery

**DOI:** 10.1155/2015/616478

**Published:** 2015-09-21

**Authors:** B. M. Wu, G. P. Williams, A. Tan, J. S. Mehta

**Affiliations:** ^1^Duke-NUS Graduate Medical School, 8 College Road, Singapore 169857; ^2^Tissue Engineering and Stem Cell Group, Singapore Eye Research Institute, The Academia, 20 College Road, Discovery Tower Level 6, Singapore 169856; ^3^Singapore National Eye Centre, 11 Third Hospital Avenue, Singapore 168751; ^4^Department of Ophthalmology, Yong Loo Lin School of Medicine, National University of Singapore, 10 Medical Drive, Singapore 117597; ^5^Ophthalmology Academic Clinical Program, Duke-NUS Graduate Medical School, 8 College Road, Singapore 169857; ^6^Department of Clinical Sciences, Duke-NUS Graduate Medical School, 8 College Road, Singapore 169857

## Abstract

The introduction of femtosecond lasers is potentially a major shift in the way we approach cataract surgery. The development of increasingly sophisticated intraocular lenses (IOLs), coupled with heightened patient expectation of high quality postsurgical visual outcomes, has generated the need for a more precise, highly reproducible and standardized method to carry out cataract operations. As femtosecond laser-assisted cataract surgery (FLACS) becomes more commonplace in surgical centers, further evaluation of the potential risks and benefits needs to be established, particularly in the medium/long term effects. Healthcare administrators will also have to weigh and balance out the financial costs of these lasers relative to the advantages they put forth. In this review, we provide an operational overview of three of five femtosecond laser platforms that are currently commercially available: the Catalys (USA), the Victus (USA), and the LDV Z8 (Switzerland).

## 1. Introduction

Cataract surgery is the most commonly performed surgical procedure, with an estimated 19 million operations performed annually [1]. The World Health Organization estimates that this number will increase to 32 million by the year 2020 as the over-65 population doubles worldwide between 2000 and 2020 [2]. In the United States, cataract affects over 22 million aged 40 and older [3], with 3 million choosing to have cataract surgery every year [4]. The Singapore Ministry of Health tallied 40,292 cataract surgeries performed in the country in 2014 [5].

Cataract surgery can be traced back over 4,000 years ago to ancient Egypt [6]. During the 20th century the introduction of intraocular lens implantation and latterly the advent of small incision phacoemulsification have had profound effect on refractive and visual outcomes [7]. Despite refinements to the technique, procedure has remained largely unchanged over the past two decades, including four main steps, such as (1) corneal incision, (2) anterior capsulotomy, (3) removal of cataractous lens, and (4) the replacement with an intraocular lens (IOL) to restore vision.

The recent advent of multifocal and toric intraocular lenses to overcome presbyopia and astigmatism, respectively, coupled with the increasing patient expectation for high quality unaided postsurgical vision, has driven scientists and clinicians alike to look toward laser technology for breakthroughs in their surgical approach [8]. This new generation of premium intraocular lenses depends on precise centration within the capsular bag for optimal performance, and anterior capsulotomy size and circularity have therefore become a key determinant to positioning and performance [9–11]. Current manual anterior capsulorhexis can present several obstacles, including dependency on surgical experience to provide consistency in producing the necessary precision, accuracy, and standardization. It has been advocated that this can be circumvented with advanced laser technology [12, 13].

The employment of lasers in cataract surgery is well established. Krasnov first described the process of “laser-phakopuncture” with the use of Q-switched lasers that operated in the nano-/picoseconds range [14]. The erbium:YAG laser was tested in 1987 on porcine eyes for capsulotomy and photoablation of the lens cortex, successfully ablating ocular tissue with minimal thermal damage [15]. It is unclear as to the specific reasons why the YAG laser never gained popularity in lens fragmentation, but one can reasonably postulate that the time required to deliver sequential shots with the YAG may be one practical deterrent; and its laser energy in the microjoule range posing potential risks for surrounding tissue damage may be another deterrent.

The femtosecond laser relies on the generation of a femtosecond of energy, to cause photodisruption, transforming tissue into plasma [16], which then rapidly expands to create microcavitation bubbles and acoustic shock waves which results in morphological tissues changes [17]. In place of traditional mechanical devices, the laser first assists in the fragmentation of the cataractous lens with the application of a number of laser pulses in a predetermined pattern; it then accurately creates a near-perfect round anterior capsulotomy with a set diameter and finally generates a small incision into the peripheral cornea for lens removal and replacement with an IOL.

The femtosecond laser's wavelength is in the near-infrared spectrum, which is not absorbed by optically clear tissues at low power densities [18, 19]. The employment of ultrafast, 10^−15^ seconds, pulses allows for far smaller amounts of energy to be used while maintaining similar power output. The benefit of these features is that delicate adjacent tissues are spared from collateral damage [20, 21].

Femtosecond lasers have been employed in ophthalmic surgery since 2001, with most widespread application in corneal and refractive surgery [22, 23]. These areas include LASIK flap creation, astigmatic keratotomy [24], channel creation for intrastromal corneal ring segment implantation [25], and lenticule extraction [26] such as smile-incision lenticule extraction (SMILE) [27]. Furthermore, several applications in corneal transplantation have been adopted, primarily through the creation of bespoke edges, such as zig-zag, mushroom, and top hat configurations [28]. It has been suggested that these edges may hasten wound healing, expedite the removal of sutures, and in turn facilitate more rapid visual recovery for penetrating keratoplasty and anterior lamellar procedures [29, 30]. Long-term data and randomized clinical trials however are lacking. Descemet stripping automated anterior lamellar keratoplasty (DSAEK) is also a technique where femtosecond lasers have been employed in an attempt to improve outcome. Currently creating ultrathin cuts by an anterior approach has proven difficult to achieve consistently and modifications of inverse cutting with low energy systems may provide the best option for achieving ultrathin DSAEK. Future developments including the optimization of real-time imaging may facilitate accurate lamellar dissections but currently this has not been optimized.

Nagy and colleagues first documented the use of femtosecond lasers in cataract surgery in 2008 [31]. The FDA subsequently approved the use of femtosecond lasers in anterior capsulotomy and lens fragmentation in 2010. Currently there are five commercial femtosecond laser platforms available. The LenSx by Alcon (Fort Worth, Texas, USA); the LensAR by LensAR (Orlando, Florida, USA); the Catalys by Abbott/Optimedica (North Chicago, Illinois, USA); the Victus by Bausch and Lomb (Rochester, New York, USA); and the LDV Z8 by Ziemer (Port, Switzerland). As of this writing, the first four platforms are FDA and CE approved; the Z8 is CE approved and is currently pending FDA approval.

In order to provide for better understanding of the technology and its role in the operating room and to help distinguish among the variations in the operational processes of the various laser platforms, this paper aims to provide a summary on the procedures of femtosecond laser in cataract surgery, and an objective and descriptive side-by-side comparison among the Catalys, the Victus, and the LDV Z8, three platforms that are accessible for femtosecond laser-assisted cataract surgery (FLACS) at our local institutions in Singapore.

## 2. Patient Selection

The absolute contraindications for this procedure have yet been clearly established, probably due to the lack of sufficient long-term follow-up studies. With continual improvements in the technology over the next years, any relative contraindications may also be subject to change. Nevertheless, at present, FLACS is generally not recommended for patients with high IOPs and those at risk of or suffering from advanced optic nerve damage, due to the increase in IOP induced by the procedure.

To date, the true rise in IOP during femtodocking and surgery has not been evaluated in real time. Current studies, including those addressing these effects in porcine and cadaveric eyes, have measured IOP following suction docking as causing a rise of up to 17.7 mmHg with the Catalys [32, 33], 40 mmHg with the LenSx [34], and up to 42 mmHg in patients undergoing surgery with the Victus [35] systems. Evaluation of real-time IOP assessment using intraocular cannulation devices however, is required to determine the pressure following applanation as they have been shown to cause IOP elevation to 200 mmHg in flat applanation systems for LASIK [36]. Theoretically these should be lower in liquid based interfaces but, given the length of the docking procedure (explained below) of FLACS, which lasts several minutes [35, 37], it is reasonable to postulate that patients at risk of glaucoma or other optic neuropathies may be less suitable for this surgery. A study by Darian-Smith and colleagues [38] in patients with primary open angle glaucoma showed a significant change in IOP in glaucomatous eyes using rebound tonometry with the Catalys system. The limitations regarding true dynamic change are still evident and the authors acknowledged that longer term follow-up was required. Thus, at this juncture, there may be greater safety margin in selecting patients with healthy optic nerves.

Another potentially difficult scenario in FLACS is the recognition of a subluxated lens or known zonular weakness. Grewal et al. [39] have shown that FLACS can be successfully undertaken in the presence of a traumatic subluxated lens and may represent potential use where minimal manipulation is required. A pupil that cannot be pharmalogically dilated can also be a relative contraindication for FLACS. But this may be overcome with Malyugin rings [40].

The presence of corneal edema and/or scarring may also pose difficulties, as the laser requires an optically clear media to pass through. However, there are no reports published on such cases so far.

Furthermore, as the procedure is dependent on successful docking, patients whose orbits are too deep for applanation or are with small palpebral apertures may pose a challenge; those who are unlikely to remain still, such as children and patients with extreme claustrophobia or with underlying neurological or psychological problems and dementia, are likely to be less suitable, though the procedure could be accomplished with the aid of general anesthesia. Since patients under general anesthesia cannot maintain fixation, FLACS would be difficult to undertake with applanation laser platforms such as the Victus. Fixation issues can be overcome with liquid-interface platforms such as the Catalys and LDV Z8, however, as long as sufficient suction is maintained throughout the procedure.

## 3. Femtosecond Laser-Assisted Cataract Surgery: Procedure

### 3.1. The Application: Femtosecond Laser

#### 3.1.1. Preparation

**Figure 1:**
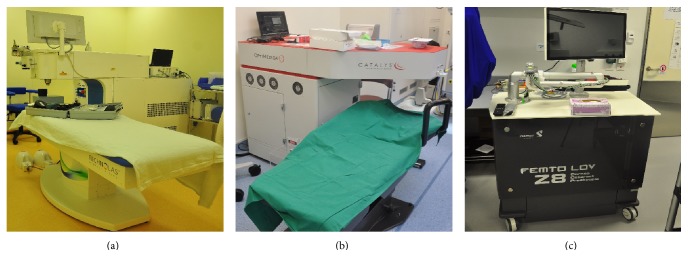
Three different femtosecond laser platforms. Victus (a), Catalys (b), and LDV Z8 (c).

Once the patient is prepared, the operating pupil dilated, and topical anesthesia applied, the patient lies down on an operating bed for the procedure. It is essential that the pupil is dilated adequately, as in conventional surgery, to facilitate successful capsulotomy and fragmentation. The Catalys and the Victus platforms come with an attached bed/table (Figures 1(a) and 1(b)), and the patient has the laser procedure performed separately, before being transferred to the operating table for completion of the relevant surgical steps, including lens removal and IOL implantation. These final surgical steps can theoretically also be completed without patient transfer, as the attached beds can be swung out diagonally, with adjustable bed heights, allowing for set up of sterile fields. Nevertheless, these two machines themselves are immobile, which may preclude their assembly in proper surgical suites with limited space. The LDV Z8 is comparably more compact and can potentially offer smaller footprint by having the platform placed on the side of a conventional operating table (Figure 1(c)).

#### 3.1.2. Docking

**Figure 2:**
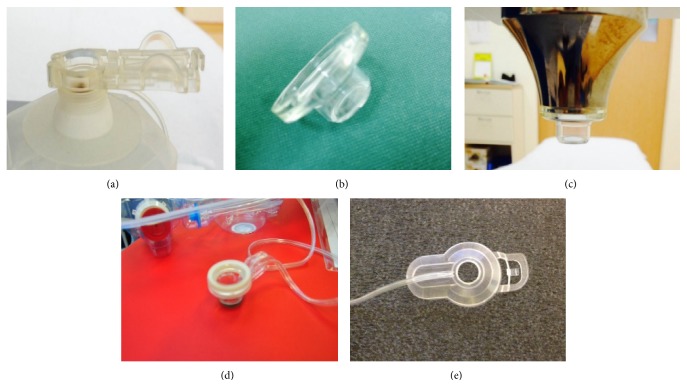
Docking devices for the three different femtosecond laser platforms. Victus suction ring (a) and curve applanation cone (b, c); Catalys suction ring (d); LDV Z8 suction ring (e).

The patient lies flat on the table, as with a standard procedure, followed by application of a suction ring with vacuum to the ocular surface (Figure 2). The surgeon determines the safe distance for the docking interface by controlling a joystick (used in the Victus and Catalys) or by manipulation of the handpiece (the Z8). It is crucial during the procedure that the patient remains steady, without eye/head movements. This suction docking process is similar to LASIK and potentially causes significant increases in intraocular pressure and may induce corneal folds and increase the risk of subconjunctival hemorrhage [32]. Some of these unwanted effects may be limited by using a liquid interface (see below).

**Figure 3:**
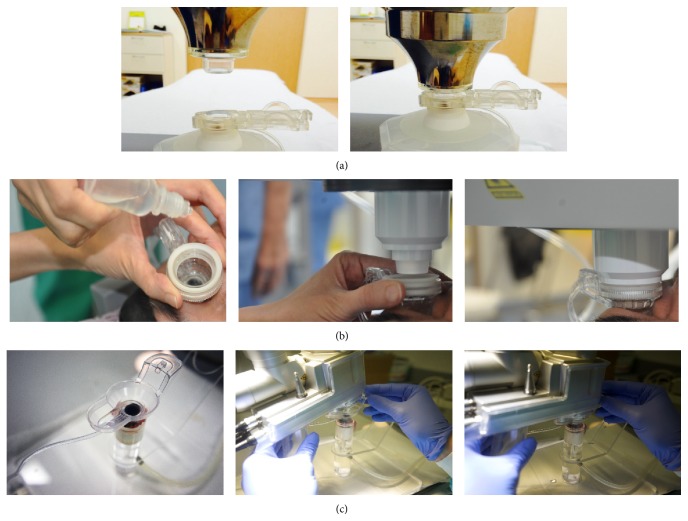
Overview of the docking processes with three femtosecond lasers. (a) Victus: suction ring applied to a sample and curved applanation cone attached to the laser head (left); laser head lowered onto the sample with vacuum on the suction ring (right). Note that, in the actual procedure, the ocular surface is moist. (b) Catalys: suction ring and application of the liquid interface (left); laser head lowered onto the ocular surface with vacuum on the suction ring (middle, right). (c) LDV Z8: suction ring and application of the liquid interface (left); manual docking of the laser handpiece with vacuum on the suction ring (middle, right). Note that surgeon's gentle support of the laser handpiece is needed throughout the procedure.

Each platform has developed its own unique interface to minimize the aforementioned problems. The Victus and the Catalys were both introduced in late 2011. The Victus employs a curved applanation device with a vacuum suction ring to facilitate adherence to the ocular surface (Figures 2(a), 2(b), 2(c), and 3(a)). If corneal folds appear, the applanation will need to be adjusted. The Catalys features a liquid interface that is nonapplanating (meaning that there is no direct contact between the laser head and the patient's ocular surface) and uses a two-piece process, with a suction ring followed by application of liquid prior to the laser head docking (Figures 2(d) and 3(b)). The LDV Z8 was commercialized in 2013 and features a similar docking process as the Catalys, first with the application of a vacuum suction ring and then liquid, but is followed instead by manual docking of the laser handpiece by the surgeon with gentle support throughout the treatment process (Figures 2(e) and 3(c)). On all three systems, the vacuum on the suction rings is automatically generated by the laser machines. Once optimal pressure has been generated to facilitate adherence of the suction ring to the ocular surface, a sound and a flash are signaled by the lasers.

#### 3.1.3. Imaging

**Figure 4:**
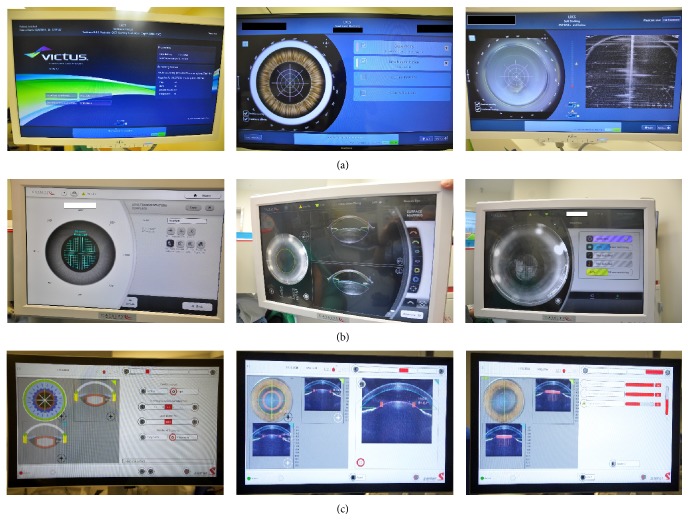
Screen shots of three femtosecond laser platforms' user interfaces. (a) Victus: (left) menu screen; (middle): selection options for FLACS procedure; (right) OCT imaging step. (b) Catalys: (left) pattern selection for lens fragmentation; (middle) OCT lens imaging; (right) FLACS progression bars: (purple) capsulotomy, (green) lens fragmentation, and (yellow) overall progression. (c) LDV Z8: (left) selection menu for FLACS parameters; (middle) OCT lens imaging; (right) (top red bar) vacuum suction pressure, (middle red bar) lens fragmentation progress, and (bottom red bar) capsulotomy progress.

The next step after successful suction docking is 3D imaging of the patient's anterior segment. The surgeon evaluates ocular structures imaged on a screen (Figure 4) and chooses predetermined parameters for OCT-guided cutting. Detailed visualization of the cornea, iris, iridocorneal angle, and lens (including anterior and posterior capsule) is the key to success and safety with FLACS [41], in order to successfully target the laser onto the lens. Inaccuracy at this stage increases the risk of incomplete capsulotomy, imprecise corneal incisions, damage to the iris, and posterior capsular rupture [41]. The Catalys, Victus, and Z8 all employ Fourier-domain OCT with high-definition imaging to map the ocular structures. The Victus further offers real-time imaging throughout the treatment process (Table 1).

#### 3.1.4. Lens Fragmentation and Anterior Capsulotomy

After ocular structures are mapped and the surgeon confirms the images, all three platforms are programmed to detect safety margins prior to laser induced lens fragmentation. The Victus has an adjustable safety margin of 0.7 mm and 1.0 mm for the anterior and posterior capsules, respectively. A default setting of 0.5 mm is set for the Catalys but is adjustable between 0.2 mm and 1.0 mm. The margins for Z8 are defaulted at 0.6 mm but can be adjusted from 0.4 mm to 1.5 mm to both the anterior and posterior capsules (Table 1).

The lens fragmentation can employ a number of different patterns based on the surgeon's choice. The Catalys has grid-pattern cuts in addition to radial cuts and the Victus offers concentric “ring” cuts, radial cuts, and grid-pattern cuts, while the Z8 has a default radial cut. During this process, the Victus fires at 80 KHz with a pulse energy of 7 *μ*J; the Catalys fires at 120 KHz with pulse energy of 8–10 *μ*J; the Z8 fires at 1 MHz with pulse energy in the nanojoule range (Table 1). The total lens fragmentation duration is dependent on the grade of the cataract; the duration of cuts relative to cataract grades is still pending further studies.

The anterior capsulotomy can either precede the lens fragmentation process (as in the Victus and the Catalys) or immediately follow it (as in the Z8). The Victus operates at 80 KHz, with a pulse energy of 6.8 *μ*J, and an unspecified/undisclosed incision depth; the Catalys operates at 120 KHz, with pulse energy of 4 *μ*J, and an incision depth of 0.6 mm; the Z8 operates at 2 MHz with a pulse energy in the nJ range and an incision depth of 0.8 mm (Table 1). On observation, the capsulotomy process across the three platforms is manually timed to take approximately 3 seconds to complete.

The most commonly performed capsulotomy size is 5.0 mm. Smaller capsulotomies can be performed, as long as there is a safe distance of 1.0 mm from the capsulotomy edge to the pupil margin. The minimum required pupil sizes for the Victus and Z8 to proceed with FLACS are 4.0 mm and 4.3 mm, respectively, while the Catalys has no specified minimum. It must also be noted that applanation can decrease pupil size, and laser energy has the potential to induce miosis of up to 2.0 to 3.0 mm [42]. It is possible that this may be overcome with mechanical dilation devices, such as Malyugin rings, with or without viscoelastics [40]. The use of preoperative anti-inflammatory medication may reduce the anterior chamber inflammatory response that may contribute to the miosis and is hence also advocated. Following completion of the capsulotomy, if miosis is an issue, then iris retractors may also be used.

#### 3.1.5. Duration of Complete Procedure

The laser procedural time itself, once the device is docked onto the eye, lasts no more than a few minutes. However, Lubahn et al. [43] reported an increase of 11.1 to 12.1 minutes in total surgery time, compared to conventional cataract surgery. This is mainly due to the specific FLACS procedure; for example, on the Victus system, Baig et al. [35] found the mean suction time to be 216 seconds (range 180 to 245 seconds), while Kerr et al. [44] found the suction time on the Catalys system to be 183 seconds (range 147 to 390 seconds). Pajic et al. with the LDV Z8 found that the average total duration of the FLACS procedure was 16.3 minutes (ranging from 12.5 minutes to 21.9 minutes) [45], reducing with increasing surgical experience, but there were no reports on suction time.

#### 3.1.6. After Laser

For the Catalys and Victus, the patient would then be transferred to the operating room for completion of the surgical procedure, which includes phacoemulsification of the cataract lens, its subsequent removal by irrigation and aspiration, and the final replacement with an IOL. With the LDV Z8, the whole operation from start to finish can be performed on the same operating table, with the surgeon simply pushing away the laser platform in order to complete the operation. Nagy et al. [31] found a 43% reduction in phacoemulsification power and a 51% decrease in phacoemulsification time needed following the use of femtosecond laser.

## 4. Management of Intraoperative Complications during FLACS

Complications during FLACS may occur at every stage of the procedure, and it is beyond the scope of this paper to give comprehensive management of all intraoperative surgical management. There are, however, circumstances which are germane to FLACS, including suction loss [46, 47], incomplete capsulotomy [48], incomplete lens fragmentation [48], capsular tags [46, 47] and adherence of the soft lens material to the capsule [49]. Suction loss may lead to the other complications listed, but if this occurs prior to laser operation, then cessation of the procedure and redocking of the laser must be undertaken, or conversion to manual technique for completion of the operation. The lasers perform capsulotomy prior to lens fragmentation, in order to allow gas escape. The Victus further allows real-time assessment of the laser fragmentation and the capsulotomy. The Z8 performs lens fragmentation first, followed by capsulotomy, and it takes an OCT image at the beginning of the procedure and following completion of the lens fragmentation. Hence, it is possible to abandon the FLACS procedure halfway through. Initial reports of capsular bag distension causing posterior capsule rupture from gas build-up have been reduced following alterations in fragmentation patterns and changes in energy settings [47, 49]. Careful placement of the laser fragmentation alignment is needed to avoid puncture through the capsule during fragmentation, especially in cases of small pupils. Incomplete capsulotomies may be overcome by manual completion under viscoelastic. Tags must be carefully identified and removed with acknowledgment that this represents an area of weakness that may lead to an anterior capsular tear during phacoemulsification or removal of lens material [47]. It is possible that soft lens matter adherence following capsulotomy relates to the energy employed during FLACS. We have found this to be less of a problem with lower energy laser systems.

## 5. Discussion

While the technical specifications vary across platforms, the greatest variation appears to lie in the docking process. The Victus and Catalys both employ an intuitive joystick system to guide the lowering of the laser head onto the ocular surface. The patients observed under these two systems have tolerated this step well without complaints. The LDV Z8 requires the laser head to be handheld, and gentle manual support of it on the eye during the laser treatment is also required, a process that may require a degree of sensitivity and experience on the surgeon's part.

One major safety concern pertains to the changes in patients' intraocular pressures over the duration of the procedure, with elevated pressures potentiating the risks for such complications as retinal ischemia, optic neuropathy, and glaucoma. The Catalys and Z8 both utilize a newer liquid interface compared to the curved applanation device employed by the Victus. As discussed previously, studies so far have shown that the IOP rise induced by the Catalys is lower than in the Victus but needs further evaluation in vivo. This may prove to be advantageous in providing a greater safety margin with regard to patients' optic nerve health [50, 51]. Nevertheless, long-term follow-up studies will be needed to access whether there is a statistically significant difference. Preliminary data in our lab on the Z8 shows optimized real-time IOP of less than 36 mmHg (Comparison of intraocular pressure changes in Ziemer LDV femtosecond laser platforms with liquid or flat applanation interfaces, Williams et al., Asia-ARVO abstract 2015, 320–P30).

Compared to the Z8, the Victus and Catalys are more sizeable and less mobile. In smaller surgical suites where the laser has to be operated in a different room altogether, this would incur a larger footprint to transfer the patient. This extra step does not affect the overall surgical procedure and appears to be of minimal hassle to the patients. The final surgical steps following laser application can also be performed without the need to transfer the patient. Furthermore, given the relative sizes of the Victus and Catalys, FLACS using these two lasers can only be performed in a specified dedicated location, whereas the more compact Z8 can be mobilized to areas convenient for the surgeons and their patients.

Although the perceived benefits are promising, femtosecond lasers have yet to replace conventional surgery. A number of practical challenges pose a barrier, and these include the financial costs of the lasers [52, 53], the learning curve of surgeons [47, 54], and the lack of sufficient literature on the risk and safety profiles of the technology, owing to its infancy. To date, we could find less than five publications using PubMed in the English language literature in the last five years addressing risks and safety [55–57].

The average laser costs between $400,000 and $550,000 [53] to be acquired, excluding the service cost after the first year, which conventionally ranges from $40,000 to $50,000 per year [53]. The cost of the disposable interfaces ranges from $300 to $450 per eye [53]. The majority of cataract operations are state-funded/subsidized, and the IOLs used are typically monofocal implants. FLACS has been advocated as facilitating the use of premium IOL implants, where precise centration and positioning of the lens is a requisite for optimal visual improvement [9–11]. Aspheric IOLs, multifocal IOLs, and toric IOLs fall under this category, but, in the USA, premium lenses make up less than 15% of all IOL implants, according to a September 2011 survey of U.S. IOL surgeons by Market Scope, LLC [58]. As a result, surgeons and administrators will have to balance the financial investment in femtosecond lasers against the volume patients requesting to undergo FLACS. The costs, however, may decrease in the future, as competition amongst providers increase, as the demonstrated risks and benefits profiles become more established, and as patients become more aware of the technology [41].

In addition, an investment in FLACS training will be necessary. However, implementation of new technology and a new skill set should not replace basic surgery skills, as there may be instances where conversion to conventional practice is warranted. The concern regarding the “flattening of the learning curve” must be balanced against potential degradation of core skills [41].

So far studies have shown that the consistency and quality of anterior capsulotomies performed with femtosecond lasers have been superior to those of manual capsulorhexis [13, 47, 59, 60]; and FLACS was associated with reduced phacoemulsification time [13, 31, 61, 62] and energy [61], with an associated reduction in endothelial cell loss [62, 63]. Furthermore, the strength of the capsular bag following laser capsulotomy in porcine eyes with intact lens has also been shown to be higher than those with manual capsulorhexis performed [13, 31, 55, 61]. Nevertheless, Abell et al. [64] found that there were a statistically significant higher number of anterior capsulotomy tags in patients who underwent FLACS using the Catalys, compared to conventional surgery.

Other cross-platform parameters of relevant significance, but still lacking in scientific data, include the effects of suction loss during the procedure; the performance of lens cutting on different grades of cataracts; and the impact of corneal clarity on the laser's penetration. How to manage these factors in large cohorts of patients is mandatory in order to establish the safety of this technology.

## 6. Conclusions

Femtosecond lasers will continue to improve and evolve in the future, but very little is still known about the mid-/long-term effects of their applications in cataract operations. Further laboratory studies, randomized controlled trials, and prospective and retrospective studies will be needed before femtosecond lasers gain stronger foothold in cataract surgery.

## Figures and Tables

**Table 1:** Feature comparison across three femtosecond laser platforms.

Laser platform	Catalys	Victus	LDV Z8
Company	Abbott/Optimedica (USA)	Bausch and Lomb (USA)	Ziemer (Switzerland)

FDA approval	Arcuate incision; anterior capsulotomy; lens fragmentation	Arcuate incision; anterior capsulotomy; lens fragmentation; corneal flaps	Pending

CE Mark	Arcuate incision; anterior capsulotomy; lens fragmentation	Arcuate incision; anterior capsulotomy; lens fragmentation; corneal flap	Arcuate incision; anterior capsulotomy; lens fragmentation

Pulse frequency			
Lens fragmentation	120 KHz	80 KHz	2 MHz
Anterior capsulotomy	120 KHz	80 KHz	1 MHz

Pulse duration	600 fs	400–550 fs	Undisclosed

Pulse energy			
Lens fragmentation	3–30 *µ*J	7.0 *µ*J	<50 nJ
Anterior capsulotomy	3–30 *µ*J	6.8 *µ*J	<50 nJ

Patient-laser interface	Liquid interface (nonapplanating)	Curved lens applanation, with ocular surface bathed in saline solution	Liquid interface (nonapplanating)

Docking	Vacuum docking	Vacuum docking	Vacuum docking

IOP during suction	28.9 mmHg (baseline: 11.4 mmHg) [44]; 25.9 mmHg (baseline: 10.3 mmHg) [33]	42.1 mmHg (baseline: 25.0 mmHg) [35]	Unknown

Lens fragmentation safety margins	0.2 mm–1 mm (adjustable) 0.5 mm (default)	0.7 mm–1 mm (adjustable)	0.4 mm–1.5 mm (adjustable) 0.6 mm (default)

Lens fragmentation patterns	Grid-pattern; radial (quadrants, sextants, and octants); grid + radial	Concentric ring-like; radial (quadrants, sextants, and octants)	Radial (default: sextants)

Capsulotomy incision depth	0.6 mm	Unspecified	0.8 mm

Can create corneal flaps?	No	Yes	Yes

Imaging	3D Fourier-domain OCT	3D Fourier-domain OCT, real-time	3D Fourier-domain OCT

Integrated bed	Yes	Yes	No
